# Para Além da Aterosclerose: É Tempo de Olhar para as Válvulas na Doença Renal Crônica

**DOI:** 10.36660/abc.20250376

**Published:** 2025-08-14

**Authors:** Sofia Cabral

**Affiliations:** 1 Centro Hospitalar Universitário de Santo António Porto Portugal Centro Hospitalar Universitário de Santo António, Porto – Portugal; 2 Universidade do Porto Instituto de Ciências Biomédicas Abel Salazar Porto Portugal Instituto de Ciências Biomédicas Abel Salazar, Universidade do Porto, Porto – Portugal

**Keywords:** Doença Cardíaca Valvar, Doença Renal Crônica, Calcificação, Risco Cardiovascular

As doenças cardiovasculares (DCV) continuam sendo a principal causa de morbilidade e mortalidade entre os doentes com doença renal crônica (DRC), ultrapassando largamente o risco de progressão para estádio terminal da doença renal e sendo responsáveis por aproximadamente 40% a 50% de todos os óbitos nos estádios avançados da doença.^1 – 4^ Temos assistido a uma crescente atenção à interseção entre disfunção renal e doença valvular cardíaca (DVC), a qual desempenha um papel importante nas complicações cardiovasculares e nos desfechos adversos observados nesta população.^5 , 6^ A calcificação valvular, que afeta predominantemente as válvulas aórtica e mitral, constitui a manifestação típica da DVC neste contexto, representando um processo altamente prevalente, progressivo e de relevância prognóstica significativa, impulsionado por alterações no metabolismo mineral, pela sua interação com inflamação crônica persistente e pelo ambiente urêmico tóxico.^7 , 8^ Apesar do crescente reconhecimento do seu impacto clínico, os fundamentos biológicos deste processo permanecem não completamente compreendidos, mesmo com o surgimento de nova evidência que começa a esclarecer os mecanismos que conduzem à calcificação valvular na DRC.

Embora tenham sido identificadas várias vias mecanísticas,^9^ continuam faltando marcadores clínicos e laboratoriais robustos que permitam prever o seu desenvolvimento. Neste sentido, o estudo de Conceição et al.^10^ oferece uma contribuição valiosa para a identificação dos fatores associados à calcificação valvular, ajudando a preencher uma lacuna relevante no conhecimento atual e sublinhando a necessidade de investigação continuada sobre as suas implicações clínicas e os mecanismos subjacentes nesta população de elevado risco.

A sua revisão sistemática se baseou em vinte estudos observacionais, envolvendo 13.314 doentes de diferentes contextos geográficos, incluindo indivíduos em diálise e fora dela. A maioria dos estudos apresentava um desenho transversal, com um número mais reduzido de estudos de coorte e apenas um estudo caso-controle, refletindo o caráter predominantemente descritivo e observacional da evidência disponível. A amplitude da população analisada, aliada ao rigor metodológico adotado, oferece uma visão abrangente do corpo de evidência existente neste domínio ainda pouco explorado.

Entre os 38 fatores de risco identificados, a idade avançada (tipicamente acima dos 55 anos) foi o mais consistentemente associado à calcificação valvular, tendo sido reportado em metade dos estudos incluídos. Estes dados reforçam o conceito de que o envelhecimento constitui um determinante importante da degeneração valvular na DRC, tal como ocorre no contexto mais amplo da doença valvular associada à idade na população geral.

Importa salientar que a redução da taxa de filtração glomerular, mesmo antes de atingir o limiar diagnóstico convencional para DRC avançada, foi frequentemente associada à calcificação valvular, com vários estudos reportando associações significativas com valores inferiores a 50 ml/min/1,73 m². Estas observações desafiam a ideia de que a calcificação valvular constitui um fenómeno exclusivo dos estádios finais da doença, apontando antes para o seu início precoce ao longo da trajetória da disfunção renal. De igual modo, entre os doentes em terapêutica de substituição renal, maiores durações de hemodiálise ou diálise peritoneal foram associadas a uma maior probabilidade de calcificação das válvulas aórtica e mitral, sugerindo um impacto cumulativo da exposição crônica ao ambiente urêmico.

Marcadores de inflamação crônica (proteína C reativa, interleucina-6 e fator de necrose tumoral alfa) e de desgaste proteico-energético (hipoalbuminémia) foram também recorrentemente associados à calcificação valvular, particularmente em doentes em diálise. Estas associações indicam que a inflamação e a disfunção nutricional poderão atuar como motores ativos da patologia valvular na DRC.^11^

Por fim, alterações no metabolismo mineral e hormonal, incluindo níveis séricos elevados de fósforo, produto cálcio-fósforo, hormona paratiroideia, fator de crescimento fibroblástico 23 (FGF-23) e níveis reduzidos de Klotho, emergiram como contribuintes fortes e biologicamente plausíveis para a patologia valvular. Estes dados acrescentam ao reconhecimento crescente de que a calcificação na DRC não resulta apenas do depósito passivo de minerais, entendido como a precipitação não regulada de complexos cálcio-fósforo, mas constitui antes um processo fisiopatológico ativo e orquestrado, que envolve múltiplas vias bioquímicas e afeta simultaneamente estruturas vasculares e valvulares.^12^ Para além da aterosclerose acelerada, este processo inclui padrões de calcificação que, embora não exclusivos da DRC, são mais proeminentes e clinicamente relevantes nesta população.^13^

Apesar de esta revisão sistemática fornecer uma visão abrangente do conjunto de fatores potencialmente associados à calcificação valvular na DRC, a predominância de estudos observacionais e transversais, a heterogeneidade das populações incluídas e as definições inconsistentes de desfechos limitam a robustez das conclusões e a sua aplicabilidade generalizada na prática clínica, aspectos corretamente reconhecidos pelos próprios autores. Ainda assim, o conjunto de evidència proveniente de diversas fontes oferece uma base sólida para a formulação de hipóteses e sublinha a necessidade urgente de investigação prospectiva e padronizada nesta área.

De forma mais abrangente, os dados apontam para a necessidade de uma estratégia cardiovascular proativa em nefrologia, com a avaliação sistemática do envolvimento valvular integrada na estratificação do risco e no planejamento dos cuidados a longo prazo. Em paralelo, o aprofundamento da investigação sobre os mecanismos subjacentes à calcificação valvular na DRC continua sendo essencial para aproximar o conhecimento fisiopatológico da intervenção clínica.

Ao consolidar evidência dispersa num enquadramento estruturado, Conceição et al.^10^ fazem uma contribuição oportuna e relevante para a compreensão do envolvimento valvular na DRC. O seu trabalho chama a atenção para o tema frequentemente negligenciado da calcificação valvular neste contexto, uma condição cada vez mais reconhecida como um potenciador do risco cardiovascular, e destaca a sua fisiopatologia complexa e multifatorial.

À medida que cresce a consciência do impacto cardiovascular associado à DRC, o avanço na prevenção e na detecção precoce da doença valvular exigirá uma colaboração sustentada entre nefrologia e cardiologia, apoiada por evidência prospectiva robusta e integrada em modelos de investigação e prática clínica em constante evolução.
