# Supplementary material for: Para Além da Aterosclerose: É Tempo de Olhar para as Válvulas na Doença Renal Crônica
Source: Arq Bras Cardiol. 2025 Aug 14;122(8):e20250376. [Article in Portuguese] doi: 10.36660/abc.20250376 (PMC12671719; doi:10.36660/abc.20250376)
Supplement: Translation

# Translation: Beyond Atherosclerosis: Mind the Valves in Chronic Kidney Disease

**DOI:** 10.36660/abc.20250376i

**Authors:** Sofia Cabral

**Affiliations:** 1 Porto Portugal Centro Hospitalar Universitário de Santo António, Porto – Portugal; 2 Porto Portugal Instituto de Ciências Biomédicas Abel Salazar, Universidade do Porto, Porto - Portugal

**Keywords:** Valvular Heart Disease, Chronic Kidney Disease, Calcification, Cardiovascular Risk

Cardiovascular disease (CVD) remains the leading cause of morbidity and mortality among patients with chronic kidney disease (CKD), far surpassing the risk of progression to end-stage renal disease, and accounting for approximately 40% to 50% of all deaths in the advanced stages.^1 – 4^Growing attention has been directed toward the intersection of renal dysfunction and valvular heart disease (VHD), which plays a major role in the cardiovascular complications and adverse outcomes observed in this population.^5 , 6^Valvular calcification, particularly affecting the aortic and mitral valves, is the hallmark manifestation of VHD in this setting, representing a highly prevalent, progressive, and prognostically significant process driven by disordered mineral metabolism, its crosstalk with persistent inflammation, and the toxic uremic milieu.^7 , 8^Despite growing acknowledgment of its clinical impact, the biological underpinnings remain incompletely understood, even as emerging evidence begins to clarify the processes driving valvular calcification in CKD.

While several mechanistic pathways have been identified,^9^robust clinical and laboratory markers that predict its development remain elusive. In this regard, the study by Conceição et al.^10^offers valuable insight into the factors associated with valvular calcification, helping to address a relevant blind spot in current knowledge and underscoring the need for continued research into its clinical implications and underlying mechanisms in this high-risk population. Their systematic review was based on twenty observational studies involving 13,314 patients from varied geographic contexts, including individuals both on and off dialysis. Most were cross-sectional in design, with fewer cohort studies and a single case-control study, reflecting the predominantly descriptive and observational nature of the available evidence. The breadth of the population analyzed, combined with the methodological rigor adopted, offers a comprehensive overview of the existing body of evidence in this still underexplored domain. Among the thirty-eight risk factors identified, advanced age (tipically above 55 years),was the most consistently associated with valvular calcification, reported in half of the studies reviewed. These findings lend further support to the concept that ageing represents a major determinant in valvular degeneration in CKD, as it does in the broader context of age-related valve disease in the general population.

Notably, a decline in gomerular filtration rate, even before reaching the conventional diagnostic threshold for advanced CKD, was frequently associated with valvular calcification, with several studies reporting significant associations at levels below 50 ml/min/1.73 m². These observations challenge the notion that valvular calcification is merely a late-stage phenomenon, instead pointing to its early onset along the trajectory of renal impairment. Similarly, among patients undergoing renal replacement therapy, longer durations of hemodialysis or peritoneal dialysis were associated with a higher likelihood of aortic and mitral valve calcification, suggesting a cumulative impact of chronic exposure to the uremic environment.

Markers of chronic inflammation (C-reactive protein, interleukin-6, and tumour necrosis factor-alpha) and protein-energy wasting (hypoalbuminemia) were also recurrently associated with valvular calcification, particularly in patients undergoing dialysis. These associations indicate that inflammation and nutritional derangement may act as active drivers of valvular pathology in CKD.^11^

Finally, disturbances in mineral and hormonal metabolism, including elevated serum phosphate, calcium-phosphate product, parathyroid hormone, fibroblast growth factor 23 (FGF-23), and reduced levels of Klotho, emerged as strong and biologically plausible contributors to valvular pathology. This adds to the growing recognition that calcification in CKD is not solely the result of passive mineral deposition, understood as the unregulated precipitation of calcium-phosphate complexes, but rather an active and orchestrated pathophysiological process involving multiple biochemical pathways and affecting both vascular and valvular structures.^12^Beyond accelerated atherosclerosis, it also encompasses calcification patterns that, while not unique to CKD, are nevertheless more prominent and clinically significant in this population.^13^

While this systematic review provides a comprehensive overview of the constellation of factors potentially associated with valvular calcification in CKD, the predominance of observational and cross-sectional designs, together with population heterogeneity and inconsistent outcome definitions, constrains the strength of the conclusions and limits their applicability across clinical settings, as correctly acknowledged by the authors. Nevertheless, the aggregation of evidence from diverse sources offers a robust basis for hypothesis generation and underscores the urgent need for prospective, standardized research in this area. Broadly, the findings call for a proactive cardiovascular strategy in nephrology, with routine evaluation of valvular involvement embedded in risk assessment and long-term care. In parallel, deepening research into the underlying mechanisms of valvular calcification in CKD remains critical to bridging the gap between pathophysiological insight and clinical intervention.

By consolidating scattered evidence into a structured framework, Conceição et al.^10^make a timely and important contribution to our understanding of valvular involvement in CKD. Their work brings renewed attention to the underappreciated burden of valvular calcification in this setting, a condition increasingly recognized as a cardiovascular risk multiplier, and draws attention to its complex, multifactorial pathophysiology.

As awareness of the cardiovascular burden associated with CKD grows, advancing prevention and early detection of valvular disease will require sustained collaboration between nephrology and cardiology, supported by robust, prospective evidence and integrated into evolving research and clinical frameworks.
